# Association Between Body Mass Index and Physical Fitness Among University Students in a Sub-Plateau Region of Gansu Province, China: A Cross-Sectional Study

**DOI:** 10.3390/life16081235

**Published:** 2026-07-26

**Authors:** Ming Chen, Linlin Zhao, Liting Yang, Mingxia Jin, Jiaqi Kong, Qin Yang

**Affiliations:** 1 School of Humanities and Social Sciences, Shanghai Lida University, Shanghai 201608, China; chenmm9122416@126.com; 2 School of Physical Education, Shanghai Normal University, Shanghai 200234, China; linlinzhao666@163.com; 3 Department of Physical Education, Tongji University, Shanghai 200092, China; 2434091@tongji.edu.cn (L.Y.); jmx021206@163.com (M.J.); 2333822@tongji.edu.cn (J.K.)

**Keywords:** body mass index, physical fitness, university students, obesity, sub-plateau region

## Abstract

While the relationship between body mass index (BMI) and physical fitness in youth often follows an inverted U-shape, populations-based data from sub-plateau settings remain scarce. This study characterized the cross-sectional BMI–fitness association among 14,744 students (9931 females) at a single university in Gansu Province, China (altitude ~1483 m). Fitness was assessed across seven components (vital capacity, speed, power, flexibility, endurance, and muscular strength/endurance) and summarized as a standardized Physical Fitness Index (PFI). Overweight/obesity prevalence was 11.2% (males 18.3%, females 7.7%)—lower than the 14.0% national average for same-aged university students. BMI displayed a monotonic inverse association with PFI; underweight and normal-weight groups performed comparably, while overweight and obese groups showed progressively lower PFI, with a stronger association in males (β = −0.46) than females (β = −0.36). The weight-normalized vital capacity index (VCWI)—a derived indicator of pulmonary function—showed the strongest inverse correlation (males r = −0.47; females r = −0.40). Associations were most pronounced for VCWI and endurance, whereas flexibility exhibited a negligible BMI-related gradient. These findings describe a cohort-specific pattern, emphasizing the need for broader investigation of regional variations, and should not be interpreted as evidence of an altitude-mediated effect.

## 1. Introduction

Overweight and obesity in adolescents constitute a pressing global public health challenge [1]. Since 1990, the global prevalence of overweight and obesity in children and adolescents has more than doubled and is projected to continue rising, thereby substantially increasing the future burden of chronic non-communicable diseases in adulthood [1].

Among emerging adults, particularly college students, body mass index (BMI) shows a consistent inverted U-shaped relationship with physical fitness indices such as the Physical Fitness Index (PFI): individuals with normal BMI exhibit the highest PFI scores, whereas those who are underweight or overweight/obese exhibit comparatively lower fitness levels [2,3,4]. In these lowland populations, the inverted U-shaped pattern has been observed across multiple fitness components, including cardiorespiratory endurance, muscular strength, and power, though the strength of the association varies by component, with cardiorespiratory fitness showing the most pronounced curvature. The BMI associated with peak fitness in these studies typically falls within the upper normal weight range (approximately 22–23 kg/m^2^), after which fitness levels decline progressively with increasing BMI [2,3,4]. Mechanistically, this pattern is commonly interpreted in relation to body composition: normal BMI reflects a balance between lean mass for force production and moderate fat mass that avoids excessive cardiorespiratory load during weight-bearing activities, whereas deviations toward either underweight or obesity shift this balance unfavorably [5]. Moreover, this relationship also exhibits sexual dimorphism, with studies observing that the detrimental effect of elevated BMI on physical fitness is generally stronger in males than in females [4]. This sex difference can be partly attributed to distinct fat distribution patterns—males tend to accumulate visceral adiposity, which more profoundly impairs cardiorespiratory efficiency and relative strength, whereas females’ predominantly subcutaneous fat deposition exerts a milder impact on movement economy [6]. Chen et al. [2] observed that underweight females performed comparably or slightly better than normal-weight peers in standing long jump, endurance running, and sit-ups, whereas underweight males had a significantly lower composite PFI. However, existing evidence supporting this consensus derives predominantly from studies conducted in plain or low-altitude regions. In such normoxic settings, the inverted U-shaped pattern has been well established, with cardiorespiratory efficiency and weight-dependent performance declining as BMI deviates from normal [2,3]. Yet these associations may not hold under hypoxic conditions, where reduced oxygen availability could fundamentally alter the BMI–fitness trade-off.

The influence of geography and altitude on the BMI-PFI relationship has gained research attention only in recent years [7]. Systematic reviews indicate that existing research on physical activity and physical fitness in university students has predominantly compared differences across dimensions such as country, institution type, and academic background, without stratifying samples by altitude, implying a data gap for mid- to high-altitude populations [8]. For example, several Chinese studies have sampled universities in eastern or central provinces, such as Shandong Province [9] and Jiangxi Province [10], which are predominantly low-altitude areas. Although the literature on altitude and fitness remains relatively limited, the existing evidence can be synthesized into three interconnected themes that collectively highlight the complex interplay between altitude, body composition, and physical performance.

First, evidence suggests that altitude influences the relationship between BMI and physical fitness, with some evidence showing non-linear (inverted U-shaped) associations where both undernutrition and overweight impair fitness, and alternative indices like the ponderal or tri-ponderal mass index may more accurately reflect body composition and fitness at moderate to high altitudes [7,11,12]. Second, studies in indigenous high-altitude populations (e.g., Tibetan youth) highlight distinct BMI-PFI patterns and body composition phenotypes, including normal-weight obesity and sex-specific differences, suggesting chronic hypoxia adaptation fundamentally shapes metabolic and fitness profiles [12,13]. Third, comparative analyses between high-/medium-altitude and lowland youths consistently find that high-altitude residents tend to have a lower prevalence of overweight and better endurance performance, despite similar or lower BMI, revealing complex interactions among altitude, morphology, and fitness [14,15].

These findings imply that the BMI–fitness relationship is not fixed but shifts with environmental oxygen availability, and that the optimal body composition phenotype for fitness may differ between altitude zones. The physiological responses to chronic hypoxic exposure provide a plausible mechanistic foundation for these observed altitude-related differences. Hypoxia activates the HIF pathway, which modulates appetite-regulating hormones and energy metabolism [16,17]. Consequently, in chronic hypoxic environments, lower body mass may reflect an adaptive optimization of oxygen cost and exercise economy. Notably, these adaptive responses exhibit sexual dimorphism, as estrogen may modulate HIF signaling and confer differential metabolic advantages in females [18,19].

In summary, current evidence emphasizes a nuanced, context-dependent interplay between altitude, BMI, body composition, and physical fitness, moving beyond simple linear assumptions [11,13,20]. However, this growing body of work has primarily examined populations residing at extreme high altitudes (e.g., >3000 m). Consequently, the BMI-PFI dynamics in sub-plateau regions (approximately 1500 m)—a transitional elevation zone defined in the Chinese sports science literature as ranging between 500 m and 3000 m, with 1500 m widely recognized as the threshold where maximal oxygen uptake begins to decline linearly with increasing altitude [21,22]—represent an under-studied elevation zone in terms of descriptive BMI–fitness data.

While the physiological mechanisms discussed above provide the contextual background for why altitude might influence the BMI–fitness relationship, the present study does not test these mechanisms directly. Rather, it adopts a descriptive approach to characterize the observed pattern in this specific cohort—specifically, to describe the BMI–fitness association in this sub-plateau setting. The objectives are: (1) to characterize the BMI distribution in this cohort; (2) to describe sex-specific associations between BMI categories and fitness components; and (3) to quantify the relationship between BMI and standardized fitness indicators as observed in this sample. Given the single-site design and the absence of a lowland comparison group, all findings are interpreted as cohort-specific and hypothesis-generating, and should not be attributed to altitude effects without direct comparative evidence.

## 2. Methods

### 2.1. Study Design and Participants

This single-site cross-sectional study analyzed routine physical fitness test data collected between 2018 and 2020 from Hexi University, located in Ganzhou District, Zhangye City, Gansu Province, where the elevation ranges from approximately 1400 m to 2000 m above sea level [23]. The campus terrain is predominantly flat, with no significant intra-campus topographic variation that would affect the standardization of fitness testing conditions. The study cohort represents an exhaustive administrative inclusion of all eligible students during the study period, rather than a convenience sample. All registered full-time undergraduate students were required to complete the annual fitness assessments as part of the university’s physical education curriculum. Students who were absent or excused due to medical reasons or other valid circumstances were scheduled for make-up tests within the same testing window. The final dataset only includes students who completed all mandatory tests, as specified in the inclusion criteria. The dataset contains unique students only, without repeated annual measurements from the same individuals. The observed sex distribution (females: 67.4%; males: 32.6%) reflects the actual demographic composition of the participating university. Hexi University is a comprehensive institution with a strong historical focus on teacher education—a disciplinary orientation that traditionally attracts a higher proportion of female students in China. Official records confirm that female students constituted approximately 70% of the 2019 freshman cohort and 64.7% of the 2021 freshman cohort, with our sample’s 67.4% falling within this consistent range. Consequently, the sex imbalance in our sample is attributable to the institution’s demographic reality rather than to selection bias in data collection. Inclusion criteria were as follows: (1) Registered full-time undergraduate students. (2) Had completed at least one full academic semester (≥3 months) at the university, a duration chosen based on two considerations: physiologically, acclimatisation to altitude (biochemical changes) begins at approximately 1500 m [21,24], and a minimum of 3 weeks at ≥1500 m is required for effective physiological adaptation, with complete haematological stabilisation requiring longer periods (>7 months at moderate altitude) [24,25]. Practically, in the Chinese university system, each academic year comprises two semesters (September–December and March–June), and routine physical fitness testing is typically conducted during the last month of each semester (November–December and May–June). The 3-month threshold thus corresponds to the interval between semester commencement and the testing window, ensuring a uniform residency period for all participants; this criterion was applied uniformly to all students regardless of their origin—whether they were lifelong residents of Gansu Province or had recently relocated from other regions—to ensure a consistent baseline for environmental exposure duration. (3) Completed all mandatory tests of the National Student Physical Health Standard (2014 Revision) with full records of height, weight, and each fitness component [26]. (4) Free from severe organic diseases, physical disabilities, or psychiatric conditions that could affect test participation or performance. Exclusion criteria comprised: (1) missing essential demographic variables (age, sex) or any key anthropometric/fitness measurement; (2) inability to complete one or more tests due to acute illness, injury, or other temporary conditions; (3) postgraduate, international, or part-time students (to maintain a homogeneous undergraduate cohort); (4) any condition judged by the investigators to compromise test safety or data validity.

Postgraduate students were excluded because their age range (>22 years) and lifestyle patterns differ substantially from the undergraduate population. International students were excluded to avoid confounding by different baseline fitness levels, nutritional habits, and ethnic backgrounds. Part-time students were excluded due to their non-continuous campus residence, which would preclude the uniform 3-month residency criterion. These exclusions limit the generalizability of our findings to the full university population, but strengthen internal validity by ensuring a relatively homogeneous undergraduate cohort.

**Figure 1:**
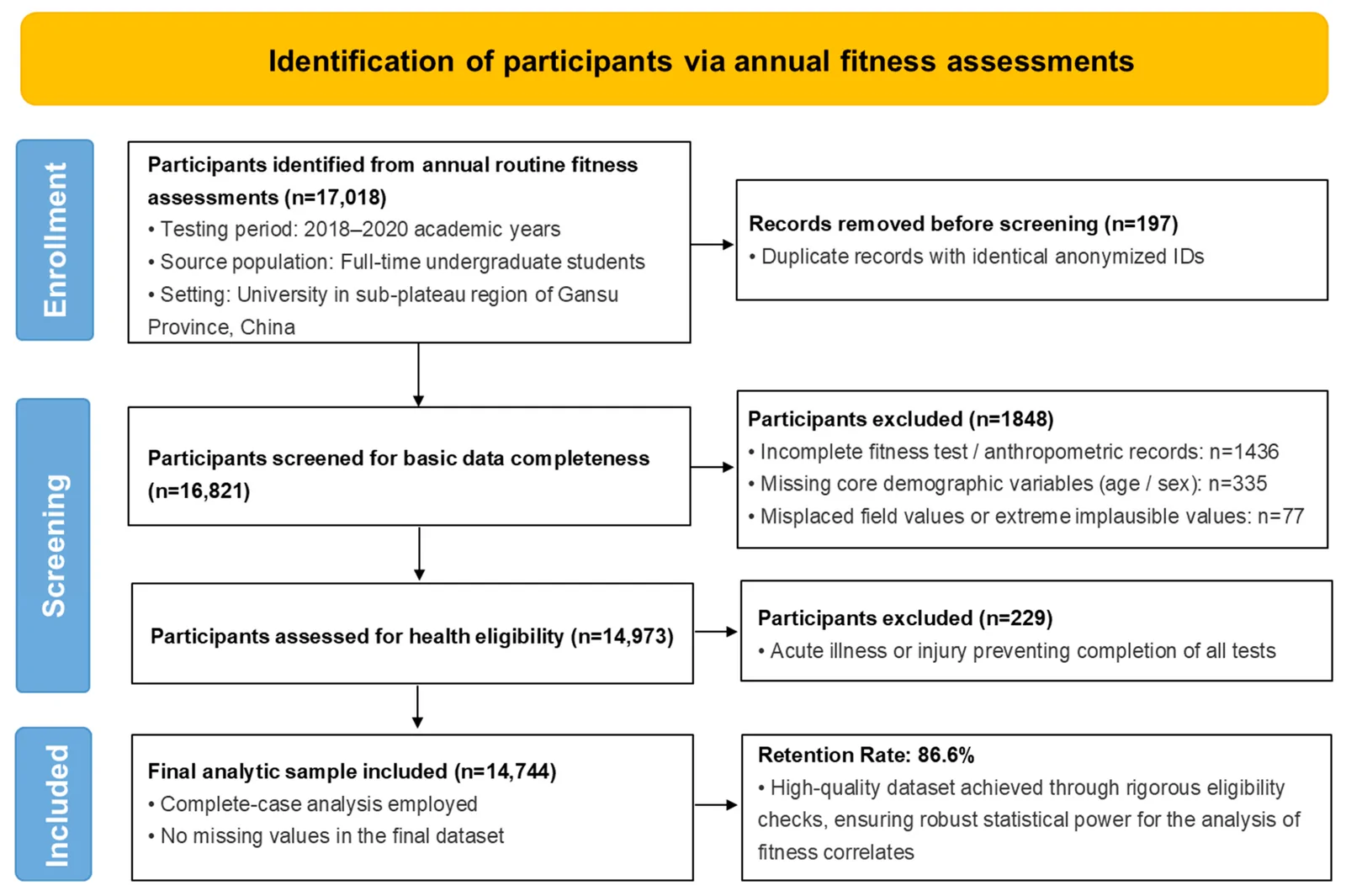
Flow diagram of participant selection.

The total eligible population during the 2018–2020 testing period comprised 17,018 full-time undergraduate students. Of these, 2077 (12.2%) were excluded, and the remaining 14,744 students (86.6%) constituted the final analytic sample (Figure 1). Because inclusion required complete data on all variables, the final dataset contained no missing values; consequently, a complete-case analysis was employed and no imputation was required. Information on excluded students was unavailable for comparative analysis due to the anonymized nature of the data; this limitation is noted in Section 4.6.

For the ethics statement, this study was conducted in accordance with the Declaration of Helsinki and was approved by the Institutional Ethics Committee of Tongji University (Approval No.: tjdxsr043). The requirement for individual informed consent was waived due to the retrospective, anonymized nature of the data obtained from routine fitness assessments.

### 2.2. Measurements and Procedures

All measurements were conducted by trained university physical education staff, with each test item administered by a fixed examiner to ensure consistency. To ensure data quality, the following procedures were implemented: (1) all examiners received uniform training prior to each testing session and followed standardized verbal instructions for each test item; (2) the same equipment was used across all testing sessions, with equipment calibrated before each session; (3) pull-up bars were standardized at a fixed height; (4) for the sit-and-reach test, examiners were trained to apply consistent pressure and scoring criteria, and the better of two trials was recorded to account for variability; (5) all tests were conducted during the same testing windows (May–June and November–December), with indoor tests performed in the morning and outdoor tests in the afternoon to minimize circadian effects; (6) participants performed standardized warm-up exercises (5 min of light aerobic activity and dynamic stretching) prior to each test. Tests were completed over two consecutive days, with non-fatiguing tests (anthropometric measurements, vital capacity, sit-and-reach) conducted on the first day and physically demanding tests (50-m sprint, standing long jump, endurance run, and pull-ups/sit-ups) on the second day to minimize fatigue effects. All fitness tests were conducted during two annual testing windows (May–June and November–December), corresponding to the end of each semester, with participants tested in batches by semester. Indoor tests—including anthropometric measurements, vital capacity, sit-and-reach, and pull-ups (males)/bent-leg sit-ups (females)—were performed in the university gymnasium, whereas outdoor tests—including the 50-m sprint, standing long jump, and 800/1000-m runs—were conducted on a standard outdoor track. All examiners received uniform training prior to each annual testing session and followed standardized verbal instructions for each test item to ensure consistent administration. The procedures strictly adhered to the National Student Physical Health Standard (2014 Revision) [26] and incorporated methods drawn from authoritative publications by leading domestic scholars [27].

#### 2.2.1. Anthropometric Measures

Height and weight were measured using a stadiometer (CSTFSG3000; Tongfang Health Technology (Beijing) Co., Ltd., Beijing, China; accuracy 0.1 cm) and a calibrated scale (CSTFTW3000; Tongfang Health Technology (Beijing) Co., Ltd., Beijing, China; accuracy 0.1 kg), respectively. BMI was calculated as weight (kg)/height^2^ (m^2^). Participants were categorized into four groups per Chinese national standards (Table 1) [26].

#### 2.2.2. Physical Fitness Tests

Health-related fitness was evaluated via the tests summarized in Table 2. All equipment was calibrated prior to each session; participants performed adequate warm-ups, wore light clothing, and avoided strenuous activity beforehand.

Based on the above fitness tests, a composite Physical Fitness Index (PFI) was calculated to summarize overall fitness, as described below.

### 2.3. Calculation of the Physical Fitness Index

The PFI was computed as a composite Z-score based on five sex-specific fitness tests: sit-and-reach, standing long jump, pull-ups (males)/bent-leg sit-ups (females), 50-m sprint, and 800-m (females)/1000-m (males) run. Raw scores for each test were first standardized to sex-specific Z-scores using the formula Z = (X − μ)/σ, where X is the raw score, μ is the sex-specific mean, and σ is the sex-specific standard deviation. The means and standard deviations used for standardization were derived from the entire cohort (stratified by sex), not from BMI-specific subgroups, ensuring comparability of Z-scores across BMI categories. To ensure higher PFI values reflect better performance, Z-scores for timed events (50-m sprint and 800/1000-m run) were multiplied by −1. The PFI was then calculated as the sum of these five Z-scores: the original Z-scores for the three non-timed tests (sit-and-reach, standing long jump, and pull-ups/bent-leg sit-ups) plus the inverted Z-scores for the two timed tests:

PFI = Z_sit_and_reach + Z_standing_long_jump + Z_pull_ups/sit_ups + (−Z_sprint) + (−Z_endurance_run)

where the Z-scores for sprint and endurance run in the formula represent the original (untransformed) Z-scores, and the negative signs complete the single inversion. Thus, each timed test is inverted only once, and no double inversion occurs. A higher PFI reflects better overall physical fitness. This equal-weighting Z-score composite method follows the established protocol validated by Yin et al. (2025) [28] in a large-scale study of Chinese college students, and is consistent with standard practice in physical fitness research for summarizing multiple components into a single interpretable index.

Vital capacity was not included in the PFI because the PFI was designed as a composite index of motor fitness (speed, power, flexibility, endurance, and muscular strength/endurance), whereas vital capacity is a measure of pulmonary function—a distinct health-related fitness component. Although VCWI showed the strongest correlation with BMI, including it in the PFI would conflate motor fitness with pulmonary function, potentially obscuring the interpretation of the BMI–motor fitness relationship. VCWI was therefore analyzed separately as a complementary indicator of pulmonary function.

### 2.4. Statistical Analysis

Continuous variables are presented as mean ± SD and categorical variables as frequency (percentage). Group differences across BMI categories were assessed using one-way ANOVA with Bonferroni-corrected post-hoc tests, applied separately for each sex stratum with 42 pairwise comparisons per stratum (7 fitness tests × 6 pairwise contrasts), with the significance threshold adjusted to α′ = 0.05/42 ≈ 0.00119. Effect sizes were calculated as partial η^2^. Linear regression was used to examine the BMI–PFI association, with a quadratic term (BMI^2^) added to test linearity and age included as a covariate. All statistical analyses were performed using SPSS (version 26.0, IBM Corp., Armonk, NY, USA), with two-tailed *p* < 0.05 considered statistically significant for omnibus ANOVA tests. Violin plots were generated using R (version 4.0, R Foundation for Statistical Computing, Vienna, Austria) with the ggplot2 and vioplot packages. Complete pairwise comparison *p*-values (uncorrected, Bonferroni-adjusted, and Benjamini–Hochberg FDR-adjusted) are provided in Supplementary Tables.

## 3. Results

To address the study aims, we first present the characteristics and BMI distribution of the cohort, followed by sex-stratified analyses of fitness differences across BMI categories, and finally quantify the associations between BMI and standardized fitness measures.

### 3.1. Participant Characteristics

The study included 14,744 students with an overall mean (±SD) age of 19.7 ± 1.5 years (18–22 years), height of 166.5 ± 7.9 cm, weight of 57.5 ± 10.2 kg, and BMI of 20.7 ± 2.8 kg/m^2^. The sample comprised 9931 females (67.4%) and 4813 males (32.6%). This sex distribution reflects the actual demographic composition of the participating university (a teacher-education-oriented institution), as detailed in Section 2.1. Male participants had a mean age of 19.7 ± 1.4 years, height of 175.1 ± 5.5 cm, weight of 65.3 ± 11.1 kg, and BMI of 21.3 ± 3.3 kg/m^2^. Corresponding values for females were 19.8 ± 1.6 years, 162.4 ± 5.0 cm, 53.8 ± 6.9 kg, and 20.4 ± 2.5 kg/m^2^, respectively.

Overall, the numbers (proportions) of underweight, normal weight, overweight, and obesity were 1048 (7.1%), 12,051 (81.7%), 1337 (9.1%), and 308 (2.1%), respectively, resulting in a combined prevalence of overweight and obesity of 11.2%. The sex-specific distribution across BMI categories is detailed in Table 3. Although males had higher mean height, weight, and BMI than females, they also exhibited a substantially higher combined prevalence of overweight and obesity (18.3% vs. 7.7%).

### 3.2. Physical Fitness Across BMI Groups in Male Students from a Sub-Plateau Region

Table 4 presents differences in health-related physical fitness across BMI categories in males. Overall, physical fitness differed significantly among BMI groups (*p* < 0.001 for all ANOVA tests). Patterns of post hoc differences after Bonferroni correction are detailed in the table footnotes.

In male students, one-way ANOVA revealed significant differences across BMI categories for all seven fitness measures (*p* < 0.001, Table 4). Effect sizes (partial η^2^) ranged from 0.02 (sit-and-reach) to 0.19 (VCWI), with most components showing medium to large effects ($\eta_{p}^{2}$ = 0.06–0.19) and VCWI reaching a large effect ($\eta_{p}^{2}$ = 0.19). Following Bonferroni correction for multiple comparisons (α′ = 0.00119), the most robust pattern was that the Overweight and Obesity groups consistently underperformed compared with both Underweight and Normal groups across most physical fitness dimensions—notably VCWI, speed (50-m sprint), lower limb power (standing long jump), cardiorespiratory endurance (800/1000 m run), and muscular strength/endurance (pull ups in males, bent leg sit ups in females). In contrast, differences between Underweight and Normal groups were generally not significant after correction, with the exception of vital capacity, VCWI, and pull-ups (all *p* < 0.001). These findings suggest that the primary fitness disadvantage is concentrated in the overweight-to-obese range, while the overall performance levels of the underweight and normal categories were broadly comparable.

### 3.3. Physical Fitness Across BMI Groups in Female Students from a Sub-Plateau Region

Table 5 presents differences in health-related physical fitness across BMI categories in females. Overall, physical fitness differed significantly among BMI groups (*p* < 0.001 for all ANOVA tests). Patterns of post-hoc differences after Bonferroni correction are detailed in the table footnotes.

In female students, one-way ANOVA revealed significant differences across BMI categories for all seven fitness measures (*p* < 0.001, Table 5). Effect sizes (partial η^2^) ranged from 0.01 to 0.10, indicating small to moderate practical significance for most components, with VCWI reaching a moderate effect ($\eta_{p}^{2}$ = 0.10) and flexibility showing a small effect ($\eta_{p}^{2}$ = 0.01). Following Bonferroni correction for multiple comparisons (α′ = 0.00119), the Overweight and Obesity groups consistently underperformed compared with both Underweight and Normal groups across most fitness measures, with the majority of these between-group comparisons reaching significance after Bonferroni correction. By contrast, differences between Underweight and Normal groups were generally not significant after correction, with the exception of vital capacity and VCWI, both of which showed significant differences (*p* < 0.001). Notably, differences between Overweight and Obesity groups were largely non-significant after correction across all measures except VCWI, which remained significant (*p* < 0.001). This pattern suggests a less distinct fitness gradient between overweight and obese females compared with males. This sex difference is further explored in the Discussion (Section 4.4) in the context of the sex-specific regression analyses. 

In summary, the association between BMI categories and physical fitness followed a consistent pattern in both male and female students from the sub-plateau region. Across most fitness measures, Underweight and Normal groups performed similarly, with no consistent statistically significant differences after Bonferroni correction. In contrast, the Overweight and Obesity groups consistently underperformed compared with both Underweight and Normal groups across most fitness measures, particularly for VCWI, standing long jump, and 800-m/1000-m run, where all pairwise comparisons between normal/underweight and overweight/obese categories were significant after Bonferroni correction. This pattern indicates that the primary fitness disadvantage is concentrated in the overweight-to-obese range, rather than reflecting a continuous linear decline across all BMI categories. The most pronounced and consistent gradient was observed for VCWI, which showed significant differences between every adjacent BMI category (all *p* < 0.00119). Notably, in females, differences between Overweight and Obesity groups were largely non-significant after correction across most measures, suggesting a less distinct fitness gradient in the upper BMI range compared with males.

### 3.4. Sex-Specific Linear Regression of BMI and PFI

To quantify the direction and magnitude of the BMI-PFI association, we performed linear regression analysis with results presented in Table 6. For both sexes, BMI showed a statistically significant negative association with PFI (both *p* < 0.001). The regression models yielded BMI coefficients of −0.46 (SE = 0.01; 95% CI: −0.49 to −0.43) for males and −0.36 (SE = 0.01; 95% CI: −0.38 to −0.34) for females, indicating that each 1-standard-unit increase in BMI corresponds to a 0.46-unit decrease in PFI for males and a 0.36-unit decrease for females. The R^2^ values were 0.04 for males and 0.03 for females.

To further validate the robustness of the BMI-PFI association, we tested the linearity assumption by including a quadratic term (BMI^2^) in the regression model. The quadratic term was statistically significant for both sexes (*p* < 0.001), indicating a detectable deviation from a purely linear shape. Specifically, the uncentered quadratic model yielded BMI^2^ coefficients of −0.002 for males and −0.001 for females. The BMI at which PFI would be theoretically maximized—calculated as BMI_max = −β_1_/(2β_2_)—yielded physiologically impossible negative values for both sexes (−110 kg/m^2^ for males and −175 kg/m^2^ for females), indicating that within the observed BMI range (approximately 14–28 kg/m^2^), the quadratic model does not predict an interior maximum. Instead, PFI declines monotonically across the observed BMI spectrum, confirming that the relationship is monotonic (PFI declines continuously with increasing BMI) rather than inverted U-shaped. We report both the linear and quadratic models for transparency, while presenting the linear model as the primary result for parsimony.

To further illustrate this pattern, we calculated predicted PFI values across the BMI continuum using the sex-specific regression models (Table 6) and the BMI standard deviations from Table 3 (SD_male = 3.3 kg/m^2^; SD_female = 2.5 kg/m^2^). For males, predicted PFI decreased from approximately 0.55 SD above the mean at BMI = 18.5 to near the mean at BMI = 22.5, and further declined to approximately 0.55 SD below the mean at BMI = 26.5. For females, the corresponding decreases were approximately 0.58 SD across each 4-unit BMI increment. This continuous monotonic descent, with no peak within the normal-weight range, confirmed a monotonic inverse pattern, differing from the inverted U-shape reported in lowland populations, which is defined by an optimal fitness range in the normal-weight category.

We next examined the influence of age by fitting full regression models including age, sex, BMI, BMI^2^, and the BMI × sex interaction term (Table 7), as well as sex-stratified models (Table 8). In the overall model (Table 7), BMI (standardized β = −0.172, *p* < 0.001) was the strongest independent predictor of PFI, followed by age (β = 0.043, *p* < 0.001); the quadratic term (β = −0.037, *p* < 0.001) and the interaction term (β = 0.032, *p* < 0.001) both remained significant. In the sex-stratified models (Table 8), BMI coefficients were −0.312 for males (*p* < 0.001) and −0.241 for females (*p* < 0.001), with the quadratic term remaining significant in both sexes (males: B = −0.024, *p* < 0.001; females: B = −0.015, *p* = 0.003). Age was a significant positive predictor in both sexes (both *p* < 0.001). Crucially, age adjustment did not materially alter the BMI coefficients compared with the unadjusted estimates (Table 6), and the adjusted R^2^ values (0.041 for males, 0.029 for females) remained consistent with the unadjusted models. These results confirm that the monotonic inverse BMI–PFI association is robust and not materially confounded by age.

### 3.5. Sex-Stratified PFI Distribution

**Figure 2:**
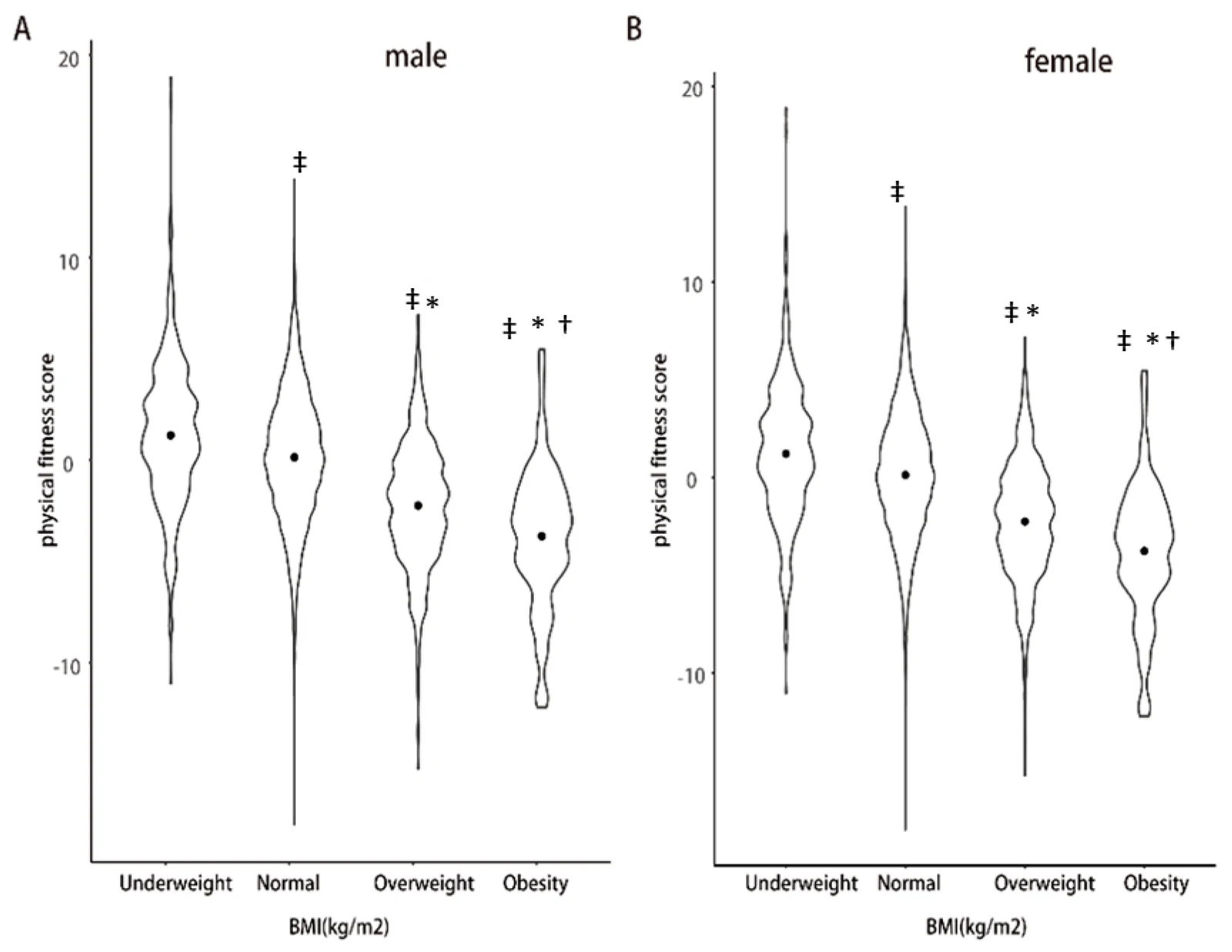
Sex-stratified violin plots of standardized Physical Fitness Index (PFI) scores across body mass index (BMI) categories. Violin plots (generated using R version 4.0) depict PFI distributions separately for males (**A**) and females (**B**), across four BMI groups: Underweight, Normal, Overweight, and Obesity. PFI scores were standardized to a mean of 0 and standard deviation of 1 prior to visualization. Within each violin, the box represents the interquartile range (IQR; 25th to 75th percentiles), with the median indicated by a horizontal line. Whiskers extend to the most extreme data points within 1.5 × IQR; points beyond the whiskers are plotted individually as outliers. Sample sizes: Underweight (males: *n* = 430; females: *n* = 618), Normal (males: *n* = 3501; females: *n* = 8550), Overweight (males: *n* = 684; females: *n* = 653), Obesity (males: *n* = 198; females: *n* = 110). Group differences in PFI were evaluated via one-way ANOVA, followed by Bonferroni-corrected post-hoc pairwise comparisons (α′ = 0.05/6 = 0.0083 for comparisons within each sex). Significance symbols: ‡ indicates significant difference from the Underweight group after Bonferroni correction; * indicates significant difference from the Normal group after Bonferroni correction; † indicates significant difference from the Overweight group after Bonferroni correction. As visualized, the Underweight group exhibited the highest PFI values, while the Obesity group showed the lowest, with the Normal group occupying an intermediate position.

Figure 2 illustrates the sex-stratified PFI distributions across BMI categories. PFI progressively declined with increasing BMI, with the Obesity group showing the widest variability and the Normal group the narrowest. All intergroup differences were statistically significant after Bonferroni correction (see figure legend for symbol definitions).

### 3.6. Correlations Between BMI and Individual Fitness Indicators

Correlations between BMI and individual fitness indicators are presented in Supplementary Table S3 and visualized in Supplementary Figures. The overall pattern showed that BMI was significantly correlated with all fitness measures in both sexes. The strongest correlation was observed with VCWI (males: r = −0.47, *p* < 0.001; females: r = −0.40, *p* < 0.001), while the weakest was with sit-and-reach flexibility (males: r = −0.032, *p* = 0.025; females: r = −0.037, *p* < 0.001). All other fitness indicators exhibited intermediate, weak-to-moderate correlations (see Supplementary Table S3 for complete r and *p*-values). To further validate the BMI-VCWI association while minimizing the influence of mathematical coupling, we conducted a supplementary partial correlation analysis between BMI and raw vital capacity (VC), controlling for height and age as detailed in Supplementary Table S4. The partial correlation coefficients were r_partial = 0.208 (df = 4809, *p* < 0.001) for males and r_partial = 0.175 (df = 9927, *p* < 0.001) for females. These results indicate that after controlling for height and age, BMI remained significantly positively correlated with raw VC in both sexes, confirming that the observed inverse BMI–VCWI association reflects a genuine decline in weight-normalized pulmonary function rather than a purely statistical artifact of mathematical coupling.

To further address the potential mathematical coupling between BMI and VCWI, we performed supplementary correlation analyses using an alternative pulmonary function index that does not include body weight in its denominator—specifically, the Vital Capacity Index (VCI), defined as VC/Height^2^ (mL/m^2^). BMI showed a statistically significant positive correlation with VCI in both sexes (males: r = 0.209, *p* < 0.001, 95% CI: [0.182, 0.235]; females: r = 0.177, *p* < 0.001, 95% CI: [0.157, 0.197]). Although the magnitude of these correlations was attenuated compared with BMI–VCWI correlations—as expected given the removal of mathematical coupling—the direction and statistical significance remained consistent with the primary findings, supporting a genuine inverse relationship between BMI and pulmonary function independent of weight-normalization. The weak BMI–flexibility correlation is further explored in the Discussion (Section 4.3).

## 4. Discussion

### 4.1. Main Results

The present study provides a comprehensive analysis of the relationship between BMI and physical fitness among university students at a single university in Gansu Province, China (altitude ~1483 m). The core findings reveal several key patterns observed in this cohort. First, the BMI distribution in this cohort exhibited a distinct profile, characterized by a lower combined prevalence of overweight/obesity (11.2%) compared to many plain-based national samples, yet with a pronounced sex disparity—males showed a substantially higher rate (18.3%) than females (7.7%). Second, and most notably, whereas lowland studies commonly report an inverted U-shaped relationship, the pattern in this cohort approximated a monotonic, declining trend, with the highest overall PFI observed in the underweight group, followed by a progressive decline through the normal weight, overweight, and obesity categories. Third, this graded decline was evident across nearly all specific fitness components, with VCWI showing the most pronounced and consistent deterioration. Finally, linear regression analyses quantified a significant negative BMI-PFI association for both sexes, which was substantially stronger in males (β = −0.46) than in females (β = −0.36). Collectively, these results highlight an observed monotonic association within this specific cohort regarding the BMI–fitness relationship, where a lower BMI, even into the underweight range, is associated with superior composite physical fitness in this cohort, and where this relationship remains distinctly modulated by sex.

It should be noted that BMI explained only 3–4% of PFI variance (R^2^ = 0.04 for males, 0.03 for females), indicating that the majority of fitness variation is attributable to other factors (e.g., physical activity, dietary habits, genetics). Nonetheless, the consistent negative association across seven independent fitness indicators, with a clear dose–response gradient, supports the robustness of the observed pattern. The public health relevance lies in the cumulative effect of even modest BMI-associated fitness reductions during young adulthood, a key period for establishing lifelong health trajectories.

### 4.2. BMI Profile and Overweight/Obesity Prevalence in This University Cohort

Before discussing the potential role of altitude, it is important to note that the observed lower prevalence of overweight/obesity in this cohort (11.2%)—while directionally consistent with altitude-related patterns reported in the literature—cannot be robustly attributed to altitude per se. Several competing explanations are equally plausible and cannot be empirically distinguished from altitude effects given the available data. These include regional dietary patterns in Northwest China, the lower socioeconomic status of Gansu Province compared with coastal regions, the ethnic composition of the local population (including Hui and Tibetan groups), and institution-specific physical education requirements at Hexi University. Because we did not collect data on dietary habits, socioeconomic status, ethnicity, or institution-specific curricula, we cannot determine the relative contribution of altitude versus these regional confounders. Therefore, the following discussion of altitude-related mechanisms should be interpreted as speculative and hypothesis-generating, rather than as a data-driven conclusion.

The study sample consisted of undergraduate students who met the inclusion criterion of having resided at the university for at least one full academic semester (≥3 months) prior to testing (see Section 2.1), ensuring a consistent environmental exposure period for all participants. Their relatively settled lifestyles make them suitable for reflecting the environmental context in which this cohort resided. The mean BMI of this cohort (20.7 kg/m^2^) was comparable to the recent average reported for Chinese university students of the same age group. Notably, however, the combined prevalence of overweight/obesity (11.2%) was lower than the 2019 national data for university students (14.0%) [29]. This observed prevalence is directionally consistent with findings from studies conducted in high-altitude populations, which report that plateau residents generally exhibit a lower prevalence of obesity and distinct body composition phenotypes. For instance, a large-scale study of Peruvian children and adolescents aged 6–16 found that the prevalence of overweight among those living at high altitude (6.3%) was substantially lower than in Amazonian (18.8%) and sea-level regions (41.3%) [15]. Although that study involved higher altitudes, the gradient trend it revealed—”higher altitude, lower overweight rate”—is directionally consistent with our finding of a lower overweight/obesity rate among subplateau university students compared to the national plain-area average. However, as we lack a lowland comparison group within the same study design, we cannot determine whether this difference is attributable to altitude, regional dietary habits, socioeconomic factors, or other geographically confounded variables.

Speculatively, the lower overweight/obesity prevalence observed in this cohort could be related to the sub-plateau environment (e.g., mild hypoxia, low air pressure, cold) or to local dietary patterns, socioeconomic characteristics, or other unmeasured factors [30]. However, because we did not collect dietary intake, physical activity, or socioeconomic data, we cannot distinguish between these possibilities. The observed prevalence difference should therefore be interpreted as a descriptive finding from this specific cohort, not as evidence of an altitude-mediated effect.

### 4.3. Pattern of the BMI–Physical Fitness Relationship in This Cohort

A core finding of this study is the nearly monotonic negative correlation between BMI and physical fitness (for both the composite PFI and most individual test items). However, this association is primarily driven by differences between the Normal/Underweight groups and the Overweight/Obesity groups, while Underweight and Normal categories are largely indistinguishable across most fitness measures. This pattern suggests that the fitness disadvantage is concentrated in the higher BMI range, rather than following a continuous monotonic decline across the entire BMI spectrum [2]. Before considering potential interpretations, it is important to emphasize that our cross-sectional, single-site design cannot establish whether this difference is attributable to altitude, regional factors, or other unmeasured confounders.

As reported in Section 3.6, supplementary partial correlation and VCI analyses supported a genuine inverse BMI-pulmonary function relationship, although the physiological interpretation should remain cautious.

With these caveats in mind, one plausible hypothesis is that cardiorespiratory efficiency becomes a key limiting factor. If excess body weight contributes to reduced cardiorespiratory efficiency, any excess body weight, particularly adipose tissue, could increase the load on the cardiorespiratory system. Fat accumulation in the chest and abdomen restricts diaphragm movement and reduces thoracic compliance, while increased body weight itself elevates total oxygen demand [31]. This makes VCWI—as a weight-normalized index of pulmonary function—an extremely sensitive indicator, where even a modest increase in BMI can lead to a significant decline in its efficiency.

A second plausible hypothesis concerns movement economy. Performing activities like running and jumping requires work against one’s own body weight. A higher BMI translates to a greater oxygen cost per unit of movement [32,33], which could explain the significant decline in performance for endurance runs (1000/800 m) and speed/power events (50-m sprint, standing long jump) as BMI increases.

A third plausible hypothesis, based on metabolic adaptation, is that body composition may play a role. A study on trained adolescents found significant differences in complex lipid and fatty acid metabolism between populations residing at different altitudes, particularly in glycerophospholipid and sphingolipid metabolism—changes associated with cardiorespiratory adaptability [34]. Although reduced oxygen availability can decrease muscle mass to some extent, this reduction is typically less pronounced than fat loss, and moderate exercise can help maintain or even increase muscle mass [35,36]. In this study, the lack of statistical difference in multiple indicators between the low-BMI and normal-weight groups, coupled with the fact that their BMIs were mostly near the lower limit of the normal range, may reflect a pattern that could be related to body composition differences. However, as we did not measure body composition directly, this interpretation remains speculative. Consistent with the pattern noted above, the comparable performance between Underweight and Normal groups suggests that the physiological adaptations conferring fitness benefits are not exclusive to underweight status per se, but rather reflect the avoidance of excess adiposity that impairs cardiorespiratory and movement efficiency in the overweight-to-obese range.

An additional observation worth noting is the near-zero correlation between BMI and sit-and-reach flexibility (r = −0.032 to −0.037; Supplementary Table S3). This negligible association contrasts sharply with the moderate-to-strong correlations observed for weight-sensitive measures such as VCWI, endurance running, and pull-ups. Several factors may explain this pattern. Biologically, flexibility is primarily determined by joint structure, ligament elasticity, and muscle extensibility—factors that are largely independent of adiposity or muscle mass. Unlike cardiorespiratory or strength measures, which are directly influenced by body weight during weight-bearing activities, sit-and-reach performance reflects the mechanical properties of the musculoskeletal system rather than metabolic or cardiorespiratory status. Methodologically, the sit-and-reach test is influenced by anthropometric factors such as leg length, sitting height, and hip joint mobility, which introduce variability unrelated to BMI. Consequently, even substantial differences in body weight may not translate into meaningful differences in flexibility performance. This finding underscores the multidimensional nature of physical fitness and suggests that BMI should not be used as a proxy for overall fitness, particularly for components such as flexibility that are largely independent of body composition.

It must be emphasized that these findings should not be interpreted as advocating that “the thinner, the better.” First, BMI is an anthropometric index that does not distinguish fat mass from fat-free mass: a low BMI may reflect low muscle mass or sarcopenia, and underweight status is associated with nutritional deficiencies, reduced bone mineral density, osteoporosis, impaired immune function, and increased frailty—all of which have well-documented adverse health consequences. The observed association between lower BMI and higher PFI in this cohort does not imply causality or universal desirability of underweight status. Rather, it simply describes the pattern observed in this specific cohort and does not provide advice: lower BMI values were associated with higher PFI scores. Importantly, future studies incorporating direct body composition measures (e.g., bioelectrical impedance analysis or dual-energy X-ray absorptiometry) are needed to determine whether the observed associations are driven by adiposity, lean mass, or other physiological factors. Readers should not interpret the observed association as a recommendation to pursue underweight status. This is a descriptive finding from a specific cohort, not a health guideline, and should not be used to inform individual weight management decisions.

### 4.4. In-Depth Analysis of Sex Differences: Interaction of Biological and Sociocultural Factors

This study identified sex as a significant moderator in the BMI–physical fitness relationship, and this sex-specific pattern merits closer examination. Male students exhibited an overweight/obesity rate 2.4 times higher than females and experienced a stronger negative effect of BMI on PFI (β = −0.46 vs. −0.36). This disparity may arise from a complex interaction between biological predispositions and sociocultural contexts.

The following biological and sociocultural interpretations are based on external literature and are offered as plausible explanations for the observed sex differences. However, because we did not collect data on body composition, dietary habits, physical activity levels, or sociocultural attitudes in this study, these interpretations should be considered speculative and hypothesis-generating rather than data-driven conclusions. 

Biologically, it has been suggested that males generally have greater muscle mass and fat-free mass. Yet, in this cohort, males with higher BMI may still accumulate substantial fat, potentially exacerbating cardiorespiratory load and resulting in a more marked decline in performance on relative strength tests (e.g., pull-ups) [37]. In contrast, the higher baseline body fat percentage and distinct fat distribution in females may lead to a different adaptive response in energy storage and metabolism within this environment.

Socioculturally, evidence from other studies indicates that male university students in China are more prone to unhealthy dietary practices—characterized by higher intake of protein, fat, and sugary foods—which has been associated with elevated obesity risk [38,39]. Conversely, female students are more influenced by societal ideals of thinness and engage more frequently in active weight control [40]. While these factors may contribute to the observed gender differences, we did not directly measure dietary intake, physical activity levels, or sociocultural attitudes in this study; thus, these interpretations remain speculative and should be interpreted with caution [40]. These patterns, however, are context-dependent. International comparisons show divergent trends: while no significant gender disparity was found in the United States [41], increases in overweight and obesity were observed only among females in South Africa [42]. This underscores how localized sociocultural factors interact with biological predispositions to produce population-specific gender outcomes. However, these comparisons are intended to illustrate the diversity of sociocultural contexts rather than to establish universal conclusions from our single-site data, and should be interpreted with appropriate caution. 

Together, these distinct behavioral patterns and environmental and lifestyle factors may shape the observed gender divergence: males face higher obesity risk and associated fitness declines, whereas females exhibit lower obesity rates and more favorable metabolic profiles. In this study, physical fitness in female students was not compromised and was associated with a lower body weight load, likely reflecting combined environmental and lifestyle influences [21]. These findings underscore the need for gender-tailored health strategies. 

A notable sex-specific pattern emerged from the post-hoc comparisons: while males exhibited a clear stepwise decline from Overweight to Obesity across most fitness measures, females showed minimal and often non-significant differences between these two categories (Table 4 and Table 5). This finding aligns with the sex-specific regression results (Section 3.4), which showed a stronger BMI-PFI association in males (β = −0.46) than in females (β = −0.36). Several complementary factors may explain why the BMI–fitness gradient is less distinct in females.

Biologically, females naturally have higher body fat percentages than males, yet their fat is predominantly distributed subcutaneously rather than viscerally [43]. Visceral adiposity—more common in males—imposes greater mechanical restriction on diaphragmatic excursion and cardiac function [6], whereas subcutaneous fat exerts a less detrimental impact on movement economy and cardiorespiratory efficiency [44]. Consequently, even when overweight or obese, females may experience a less pronounced decline in weight-dependent physical performance.

Physiologically, sex differences in metabolic responses to hypoxia may also play a role. Estrogen appears to modulate hypoxia-inducible factor (HIF) signaling, potentially conferring more favorable oxygen utilization efficiency in females [45]. Furthermore, females exhibit greater reliance on fatty acid oxidation during submaximal exercise [46], which may provide a metabolic advantage under reduced oxygen availability. These sex-specific physiological responses could partially offset the performance decrements associated with higher BMI in females.

Methodologically, the sex-specific PFI uses different muscular strength tests: pull-ups for males and bent-leg sit-ups for females. Pull-ups are highly weight-dependent—excess body mass directly increases the gravitational load—whereas bent-leg sit-ups are less influenced by body weight [4]. This difference likely amplifies the BMI–fitness gradient in males while attenuating it in females, contributing to the observed attenuation of differences between overweight and obese females.

While the BMI-by-sex interaction was statistically significant, it should be noted that the sex-specific PFI includes different muscular strength tests (pull-ups for males, bent-leg sit-ups for females), which may partly influence the magnitude of the observed coefficient difference. Nevertheless, the direction of the interaction is consistent with the biological expectation that excess body mass impairs weight-dependent performance more substantially in males.

Importantly, these interpretations are constrained by the absence of direct measurements of body composition, dietary intake, physical activity, and sociocultural attitudes in our study.

### 4.5. Research Implications: Considerations for Health Promotion and Physical Education in Subplateau Regions

Our descriptive findings offer several concrete takeaways for policy and practice. The effect sizes (partial η^2^) were largest for VCWI (0.19 in males, 0.10 in females) and endurance-related measures, but minimal for flexibility ($\eta_{p}^{2}$ = 0.01–0.02). This suggests that interventions targeting cardiorespiratory and weight-dependent fitness components are most relevant to BMI-associated disparities in this population, while flexibility-focused efforts may have limited impact.

These findings align with current national policy priorities. The “Healthy China 2030” Planning Outline mandates that adolescent students engage in moderate-to-vigorous physical activity at least three times per week, while the “Basic Standards for Sports Work in Higher Education Institutions” [47,48] requires cardiopulmonary fitness training to account for ≥30% of physical education class time and assessment weight. More recently, the “Student Physical Fitness Strengthening Plan” [49] calls for universities to organize at least three sessions of intensive extracurricular physical activity per week. Our findings—particularly the graded decline in VCWI and endurance performance across BMI categories—provide empirical support for these policy directives, and the stronger BMI–fitness association observed in males (β = −0.46 vs. −0.36) suggests that gender-tailored strategies—prioritizing cardiorespiratory interventions for males and weight management education for females—may be worth considering.

The following suggestions are derived from our observational data and should be considered speculative and hypothesis-generating rather than evidence-based guidelines. Because our cross-sectional design cannot establish causality, we cannot determine whether interventions targeting BMI would improve fitness, nor did we test the effects of weight change on fitness outcomes. These suggestions therefore require validation through future interventional or longitudinal studies before they can be translated into formal recommendations.

We therefore propose the following considerations for improving student physical health initiatives at universities in similar geographic settings, drawing on the descriptive findings of this single-site cross-sectional study and the relevant literature.

First, regarding physical fitness assessment, prioritizing VCWI in routine evaluations may be worth considering, given its strong correlation with BMI and overall fitness. VCWI could serve as a potentially useful indicator for evaluating cardiorespiratory efficiency in this setting [50]. Concurrently, educational authorities may wish to explore allocating funding and expertise to research and develop regional BMI–physical fitness reference standards that better align with the physiological characteristics of subplateau areas. Such efforts, if validated, could contribute to establishing more geographically appropriate physical health assessment frameworks.

Second, it may be worth considering reforms to the content of physical education teaching and training. Physical education curricula could specifically incorporate training that enhances cardiorespiratory efficiency and muscular strength/endurance, such as high-intensity interval training, circuit training, and resistance training [51]. For students with higher BMI, exercise modalities that reduce body weight load while still effectively stimulating the cardiorespiratory system and muscles—such as swimming, cycling, and upper-body strength training—could be designed to increase their participation and exercise benefits.

Third, it may be valuable to consider implementing targeted health education and management. Health education and publicity could disseminate knowledge about the unique relationship between body weight and physical fitness in this setting. This could help guide students to develop a scientific concept of weight management, avoiding the blind pursuit of excessively low weight or the neglect of weight gain. Universities could consider establishing a cross-departmental collaboration mechanism (involving the Physical Education Department, University Hospital, Student Affairs Office, and Dining Services Center) to provide overweight/obese students with integrated “nutrition–exercise–psychology” interventions and to offer underweight students guidance on balanced nutrition and scientific muscle gain. Given the stronger BMI–fitness association observed in males, gender-tailored components within these interdisciplinary interventions may also warrant consideration.

In summary, these suggestions are intended as hypothesis-generating directions for future research and practice, not as established recommendations. Their implementation should be accompanied by rigorous evaluation to determine whether they produce the intended fitness benefits, and our findings should be validated in other sub-plateau populations before broader application.

### 4.6. Limitations and Future Perspectives

Several limitations should be acknowledged. First, the cross-sectional, single-site design precludes causal inference and cannot rule out reverse causality. Moreover, the absence of a lowland comparison group means we cannot determine whether the observed monotonic BMI–fitness pattern is attributable to the sub-plateau environment or to other geographically confounded factors; consequently, any interpretation of the BMI–fitness pattern as an ‘altitude effect’ remains speculative, and we cannot exclude the possibility that regional dietary patterns, socioeconomic status, ethnic composition, or institutional factors contribute to the observed pattern independently of altitude. Thus, our findings represent a descriptive characterization of this specific cohort. Furthermore, the single-site design limits generalizability to other sub-plateau regions (e.g., in Yunnan or Sichuan provinces) with different environmental, dietary, or demographic characteristics; multi-site replication is needed before any broader conclusions can be drawn. It is also important to note the substantial sex imbalance in our sample (67.4% female). While this distribution reflects the actual student composition of the participating teacher-education-focused university, it may limit the generalizability of our findings to university populations with markedly different sex ratios (e.g., engineering or technology-focused institutions where male students predominate). Nevertheless, because all analyses were conducted separately for each sex, the internal validity of the sex-stratified results is not compromised by the overall sex imbalance. Additionally, because the data were anonymized, we could not compare included participants with excluded students and therefore cannot assess potential selection bias. However, the direction of potential bias can be inferred from the exclusion reasons. The largest exclusion category (incomplete records, 8.4%) was likely random rather than systematic, and acute illness/injury (1.3%) is unlikely to be associated with BMI or fitness. The 3-month criterion may have excluded recent lowland arrivals, potentially biasing the sample toward more settled students and thus attenuating rather than exaggerating altitude-related effects. The large sample size and consistent BMI-PFI association across multiple fitness components (Table 4 and Table 5) suggest that any such bias is unlikely to have fundamentally altered our findings. Future longitudinal studies are needed to establish temporal precedence and clarify the direction of this association.

Second, although key anthropometric and fitness metrics were included, data on detailed dietary patterns, physical activity intensity, sedentary behavior, smoking, socioeconomic status, and ethnicity were not collected. For example, if students with healthier dietary patterns also engage in more physical activity, failing to adjust for these factors could lead to overestimation of the BMI–fitness association. Similarly, socioeconomic status may influence both dietary quality and access to physical activity facilities. This absence may introduce residual confounding; consequently, the interpretations in Section 4.2 and Section 4.4 that reference these factors remain speculative rather than data-driven conclusions. Specifically, as discussed in Section 4.2, the observed lower overweight/obesity prevalence in this cohort could equally reflect regional dietary patterns (e.g., Northwest Chinese cuisine), lower socioeconomic status in Gansu Province, ethnic composition (Hui and Tibetan populations), or institution-specific physical education requirements—factors that we cannot empirically distinguish from these environmental and institutional factors. 

Third, sex identity was not assessed in this study, as the data were derived from routine fitness assessments that recorded only biological sex, and the study therefore did not specifically include or identify transgender or sex-diverse individuals, which constrains the generalizability of the sex-specific findings to the broader student population. 

Fourth, BMI was used as the sole indicator of weight status. BMI is a proxy measure that cannot differentiate between fat mass and fat-free mass [52]. Consequently, we cannot determine whether the observed associations reflect adiposity, muscle mass, or both. Additionally, VCWI is a derived index calculated by dividing vital capacity by body weight. Because BMI also incorporates body weight (in its numerator), the observed correlation between BMI and VCWI is partly attributable to mathematical coupling. To address this, we conducted a supplementary partial correlation analysis using raw VC adjusted for height and age (see Section 3.6), which confirmed a genuine inverse relationship between BMI and weight-normalized pulmonary efficiency. The complete partial correlation results (r_partial, degrees of freedom, and *p*-values) are reported in Section 3.6 and Supplementary Table S4. However, future studies using more direct measures of cardiorespiratory fitness, such as maximal oxygen uptake, are warranted. Beyond the limitations of BMI and VCWI as individual measures, the composite PFI itself has methodological constraints. The PFI was calculated as an equally weighted Z-score sum, a common approach in physical fitness research. This assumes equal contributions from each fitness component, which may not reflect differential health relevance; moreover, Z-score composites can be influenced by component variances. Alternative weighting approaches (e.g., factor-analysis-derived or health-outcome-based weights) could be explored in future studies to assess robustness. Nevertheless, the consistency of the observed monotonic BMI-PFI association across multiple individual fitness indicators (Table 4 and Table 5) supports the robustness of our primary finding beyond the specific PFI weighting scheme.

Fifth, we acknowledge that we did not formally perform a constrained model comparison test between the quadratic model and an inverted-U-constrained model. While the physiologically negative BMI_max values derived from the quadratic model (see Section 3.4) and the negligible R^2^ improvement (ΔR^2^ < 0.001) support the monotonic interpretation within the observed BMI range, we recognize that a formal model comparison would provide additional statistical rigor. Future studies with wider BMI ranges and larger sample sizes across different altitude settings are needed to definitively test the functional form of the BMI–fitness association.

Expanding multi-center collaborations across diverse sub-plateau regions would enhance geographical representativeness. Incorporating validated measures of dietary habits, physical activity intensity, sedentary behavior, smoking, and socioeconomic status would enable more robust adjustment for potential confounders. Furthermore, adopting inclusive practices in future data collection regarding sex will help ensure that findings are relevant to all students. Finally, building on this study’s observation of the BMI–fitness relationship in this cohort, developing and validating region-specific physical fitness reference standards—incorporating indices such as the VCWI—represents an important direction for applied research and local policy-making.

## 5. Conclusions

This study observes a monotonic inverse association between BMI and physical fitness among university students in this single-site cohort from Gansu Province (altitude ~1483 m). Lower BMI was associated with better overall fitness, with a stronger association in males. However, it should be noted that BMI explained only a small proportion of PFI variance (R^2^ = 0.03–0.04), indicating that although the association was statistically significant, its practical magnitude was modest. Whether this pattern is specific to this cohort or reflects broader geographical variations remains to be determined through future multi-altitude and longitudinal studies. BMI is an imperfect proxy that does not distinguish fat mass from lean mass; accordingly, low BMI is not automatically indicative of optimal health, and this association should not be interpreted as endorsing underweight as a health goal. These findings highlight the potential need for region-specific considerations in physical fitness standards and tailored health promotion strategies, and underscore the importance of direct low-altitude comparisons in future research.

## Figures and Tables

**Table 1:** The classification standards of BMI.

Sex	Underweight	Normal	Overweight	Obesity
Male	BMI ≤ 17.8	17.8 < BMI ≤ 23.9	24 ≤ BMI ≤ 27.9	BMI ≥ 28
Female	BMI ≤ 17.1	17.1 < BMI ≤ 23.9	24 ≤ BMI ≤ 27.9	BMI ≥ 28

Note: BMI, body mass index.

**Table 2:** Components and protocols of health-related physical fitness tests.

Fitness Component	Test Item	Brief Protocol	Unit
Lung Function	Vital Capacity (VC)	Vital capacity was measured using a calibrated spirometer (Model XF495KDL).	milliliters (mL)
	Weight-normalized vital capacity index (VCWI)	Calculated as: VC (mL)/Body Weight (kg).	mL/kg
Speed	50-m sprint	Time to sprint 50-m from a standing start was recorded.	seconds (s)
Lower Limb Power	Standing long jump	Distance jumped forward from a standing position. The better of two trials was recorded.	centimeters (cm)
Flexibility	Sit-and-reach	Performed using a standard box with legs extended. The distance reached by the hands was recorded. The better of two trials was recorded.	centimeters (cm)
Cardiorespiratory Endurance	1000-m Run (M)/800-m Run (F)	Time to complete the distance was recorded.	seconds (s)
Muscular Strength/Endurance	Pull-ups (M)	The maximum number of correctly completed repetitions (chin clearing the bar) was recorded.	count
	1-min Bent-leg Sit-ups (F)	The number of correct repetitions (elbows touching knees) completed in 60 s was recorded.	count

Note: M: Male; F: Female. All procedures were performed in strict accordance with the National Student Physical Health Standard.

**Table 3:** Anthropometric and baseline characteristics of participants stratified by sex and BMI category.

Test Items	Male (*n* = 4813/32.6%)				Female (*n* = 9931/67.4%)			
	Underweight (430/8.9%)	Normal (3501/72.7%)	Overweight (684/14.2%)	Obesity (198/4.1%)	Underweight (618/6.2%)	Normal (8550/86.1%)	Overweight (653/6.6%)	Obesity (110/1.1%)
Age (year)	19.5 ± 1.3	19.7 ± 1.3	19.7 ± 1.5	19.4 ± 1.3	19.8 ± 1.6	19.8 ± 1.6	19.5 ± 1.4	19.2 ± 1.2
Height (cm)	175.2 ± 5.4	175.1 ± 5.5	175.1 ± 5.6	175.6 ± 5.9	163.2 ± 5.0	162.3 ± 4.9	161.9 ± 5.3	162.2 ± 6.1
Weight (kg)	52.3 ± 3.9	63.1 ± 6.5	78.7 ± 6.0	93.7 ± 10.5	43.9 ± 3.6	52.9 ± 5.3	66.6 ± 5.4	80.2 ± 9.2
BMI (kg/m^2^)	17 ± 0.8	20.6 ± 1.7	25.6 ± 1.1	30.4 ± 2.9	16.5 ± 1.0	20.1 ± 1.7	25.4 ± 1.1	30.5 ± 2.6

Note: Data are presented as mean ± SD. BMI, body mass index.

**Table 4:** Health-related physical fitness across BMI categories in male students (*n* = 4813).

Test Items	Underweight (1) (430/8.9%)	Normal (2) (3501/72.7%)	Overweight (3) (684/14.2%)	Obesity (4) (198/4.1%)	F	$\eta_{p}^{2}$	Post Hoc Comparison (Bonferroni-Corrected)					
							1 vs. 2	1 vs. 3	1 vs. 4	2 vs. 3	2 vs. 4	3 vs. 4
Vital capacity (ml)	3687.5 ± 736.9	4072.6 ± 757.3	4328.2 ± 874.9	4433.7 ± 827.7	73.5 ***	0.06	‡	‡	‡	‡	‡	ns
VCWI (ml/kg)	70.7 ± 14.4	64.8 ± 11.9	55.1 ± 10.4	47.6 ± 9.2	301.1 ***	0.19	‡	‡	‡	‡	‡	‡
50-m(s)	7.5 ± 0.59	7.5 ± 0.59	7.7 ± 0.62	8 ± 0.74	68.8 ***	0.06	ns	‡	‡	‡	‡	‡
Standing long jump (cm)	220.3 ± 19.1	220.7 ± 18.9	212.5 ± 19.6	202 ± 21.8	87.3 ***	0.08	ns	‡	‡	‡	‡	‡
Sit-and-reach (cm)	10.3 ± 6.6	11.4 ± 6.5	10.7 ± 6.2	9.1 ± 5.9	12.14 ***	0.02	*	ns	ns	ns	*	*
1000-m (s)	246 ± 24.4	246.1 ± 24.4	260.2 ± 25.6	281.3 ± 38.5	168.9 ***	0.12	ns	‡	‡	‡	‡	‡
Pull-up	8.1 ± 4.9	7.4 ± 5	4.2 ± 4.1	3 ± 4.1	137.9 ***	0.11	‡	‡	‡	‡	‡	*

Note: Data are presented as mean ± SD. *** *p* < 0.001 for one-way ANOVA. $\eta_{p}^{2}$ = partial eta squared (effect size). VCWI = weight-normalized vital capacity index. Post hoc comparisons adopted Bonferroni correction for 42 pairwise comparisons (α′ = 0.05/42 ≈ 0.00119). ‡ = statistically significant after Bonferroni correction (raw *p* < 0.00119); * = nominally significant only (0.00119 ≤ raw *p* < 0.05); ns = not significant (raw *p* ≥ 0.05).

**Table 5:** Health related physical fitness across BMI categories in female students (*n* = 9931).

Test Items	Underweight (1)	Normal (2)	Overweight (3)	Obesity (4)	F	$\eta_{p}^{2}$	Post Hoc Comparison (Bonferroni-Corrected)					
	(618/6.2%)	(8550/86.1%)	(653/6.6%)	(110/1.1%)			1 vs. 2	1 vs. 3	1 vs. 4	2 vs. 3	2 vs. 4	3 vs. 4
Vital capacity (ml)	2582 ± 530.8	2755 ± 566	2845.3 ± 559.6	2882.4 ± 539.5	26.8 ***	0.01	‡	‡	*	‡	ns	ns
VCWI (ml/kg)	59.5 ± 15.7	52.3 ± 10.8	42.7 ± 7.8	36.3 ± 7.3	330.2 ***	0.10	‡	‡	‡	‡	‡	‡
50-m(s)	9.4 ± 0.68	9.4 ± 0.76	9.6 ± 0.73	9.8 ± 0.78	20.6 ***	0.01	ns	‡	ns	‡	ns	ns
Standing long jump (cm)	167.5 ± 15.4	164.6 ± 14.8	160.2 ± 14.8	157.2 ± 15.8	35.7 ***	0.02	ns	‡	‡	‡	‡	ns
Sit-and-reach (cm)	13.4 ± 6.2	13.6 ± 5.8	12.2 ± 5.5	11.9 ± 5.7	14.7 ***	0.01	*	ns	ns	*	*	ns
800-m (s)	237.1 ± 22.3	239 ± 22.9	251.2 ± 22.7	257.3 ± 30.5	81.7 ***	0.03	ns	‡	‡	‡	‡	ns
Bent leg sit up	35 ± 8.9	33.5 ± 8.5	31.4 ± 8.1	29.8 ± 8.7	25.9 ***	0.01	ns	ns	ns	ns	ns	ns

Note: Data are presented as mean ± SD. *** *p* < 0.001 for one-way ANOVA. $\eta_{p}^{2}$ = partial eta squared (effect size). VCWI = weight-normalized vital capacity index. Post hoc comparisons adopted Bonferroni correction for 42 pairwise comparisons (α′ = 0.05/42 ≈ 0.00119). ‡ = statistically significant after Bonferroni correction (raw *p* < 0.00119); * = nominally significant only (0.00119 ≤ raw *p* < 0.05); ns = not significant (raw *p* ≥ 0.05).

**Table 6:** Associations of body mass index (BMI) with physical fitness index (PFI).

Outcome	BMI Coefficients (β)	Std. Error	t Value	*p*	R^2^	95% CI
PFI (male)	−0.46	0.01	−33.95	<0.01	0.04	[−0.49, −0.43]
PFI (female)	−0.36	0.01	−28.5	<0.01	0.03	[−0.38, −0.34]

Note: PFI was computed using sex-specific Z-scores (mean = 0, SD = 1 within each sex), meaning that standardization and PFI calculation were performed independently for males and females. All models were unadjusted for age; age-adjusted results are presented in Table 7 and Table 8. Because PFI is a sum of Z-scores, the intercept is not biologically interpretable and is therefore omitted from the table. β = standardized regression coefficient. R^2^ = coefficient of determination. 95% CI = 95% confidence interval for β. Data were derived from the study cohort (males: *n* = 4813; females: *n* = 9931).

**Table 7:** Full results of the overall multivariable linear regression model (full sample, *n* = 14,744).

Variable	Unstandardized B	Std. Error	Standardized β	t Value	*p* Value
Intercept	−0.214	0.102	—	−2.10	0.036
Age (years)	0.127	0.011	0.043	11.55	<0.001
Sex (ref = Male)	0.382	0.087	0.029	4.39	<0.001
BMI	−0.286	0.015	−0.172	−19.07	<0.001
BMI^2^	−0.019	0.004	−0.037	−4.75	<0.001
BMI × Sex	0.073	0.019	0.032	3.84	<0.001
Model fit	Adjusted R^2^ = 0.038	F (5, 14,738) = 116.23			<0.001

Note: Dependent variable = Physical Fitness Index (PFI). Sex coded as dummy variable (0 = male, 1 = female). BMI and BMI^2^ were grand-mean centered to reduce multicollinearity.

**Table 8:** Full results of sex-stratified linear regression models (aligned with Table 4 and Table 5).

Variable	Male Students (*n* = 4813)						Female Students (*n* = 9931)			
	Unstandardized B	Std. Error	Standardized β	t Value	*p* Value	Unstandardized B	Std. Error	Standardized β	t Value	*p* Value
Intercept	−0.187	0.156	—	−1.20	0.23	−0.163	0.129	—	−1.26	0.207
Age	0.142	0.018	0.048	7.89	<0.001	0.118	0.013	0.041	9.08	<0.001
BMI	−0.312	0.022	−0.191	−14.18	<0.001	−0.241	0.019	−0.158	−12.68	<0.001
BMI^2^	−0.024	0.006	−0.041	−4.00	<0.001	−0.015	0.005	−0.027	−3.00	0.003
Model fit	Adjusted R^2^ = 0.041	F (3, 4809) = 68.74	—	—	<0.001	Adjusted R^2^ = 0.029	F (3, 9927) = 98.51	—	—	<0.001

Note. Dependent variable = Physical Fitness Index (PFI). BMI and BMI^2^ were group-mean centered within each sex stratum. Standardized β coefficients are calculated separately within each sex sample for within-group relative contribution comparison only, and are not intended for direct cross-sex comparison.

## Data Availability

The full de-identified dataset and statistical code is available upon reasonable request from the corresponding author. The data presented in this study are not publicly available due to institutional policy (Tongji University) and the limitations of the informed consent form, under which participants were assured that their data would only be used for internal fitness assessment and anonymized research purposes. The full anonymized dataset is available on reasonable request from the corresponding author (yangqinsus@163.com), subject to institutional data access approval and appropriate data transfer agreements.

## Supplementary Materials

The following supporting information can be downloaded at: https://www.mdpi.com/article/10.3390/life16081235/s1. The following supplementary materials are available in two separate Word files: Supplementary Tables (one file containing Tables S1–S4): Supplementary Table S1. Full pairwise comparison *p*-values for male students (42 comparisons total, aligned with Table 4). Supplementary Table S2. Full pairwise comparison *p*-values for female students (42 comparisons total, aligned with Table 5). Supplementary Table S3. Pearson correlation coefficients (r) between BMI and individual fitness indicators, stratified by sex. Supplementary Table S4. Partial correlation coefficients between BMI and raw vital capacity (VC), controlling for height and age, stratified by sex. Supplementary Figures (one file containing Figures S1 and S2): Supplementary Figure S1. Correlations between body mass index (BMI) and standardized physical fitness indicators in male students. Data for each fitness indicator were standardized (z-scores; mean = 0, SD = 1). Pearson correlation coefficients (r) and *p*-values are displayed for each BMI-indicator pair. Supplementary Figure S2. Correlations between body mass index (BMI) and standardized physical fitness indicators in female students. Data for each fitness indicator were standardized (z-scores; mean = 0, SD = 1). Pearson correlation coefficients (r) and *p*-values are displayed for each BMI-indicator pair.

## Abbreviations

ANOVA	Analysis of Variance
BMI	Body Mass Index
PFI	Physical Fitness Index
SD	Standard Deviation
VC	Vital Capacity
VCWI	Weight-normalized Vital Capacity Index
