# Supplementary material for: Association Between Body Mass Index and Physical Fitness Among University Students in a Sub-Plateau Region of Gansu Province, China: A Cross-Sectional Study
Source: Life (Basel). 2026 Jul 26;16(8):1235. doi: 10.3390/life16081235 (PMC13514759; doi:10.3390/life16081235)
Supplement: Supplementary file 1 [file life-16-01235-s001.zip › life-4378054-supplementary-Figures S1 and S2.pdf]

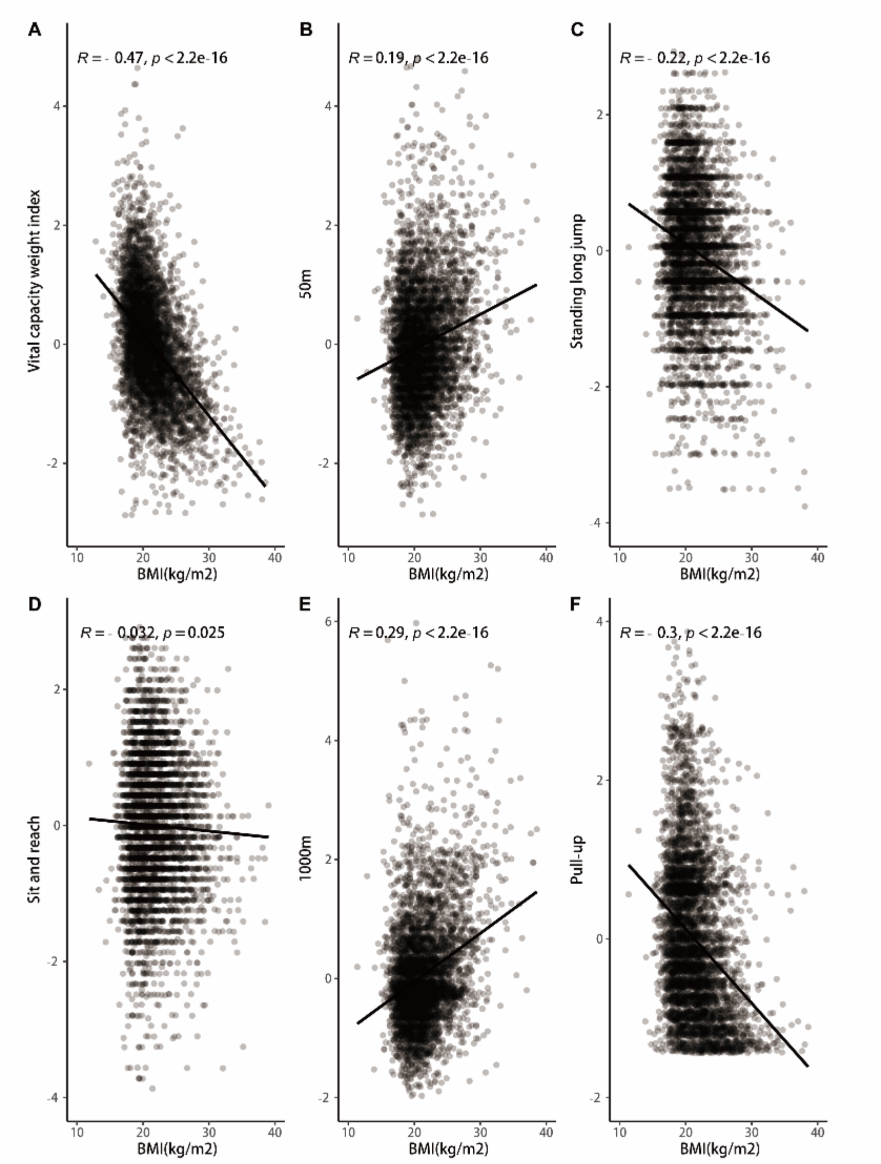

Supplementary Figure S1. Correlations between body mass index (BMI) and standardized physical fitness indicators in male students. Data for each fitness indicator were standardized (z-scores; mean = 0, SD = 1). Pearson correlation coefficients (r) and p-values are displayed for each BMI-indicator pair.

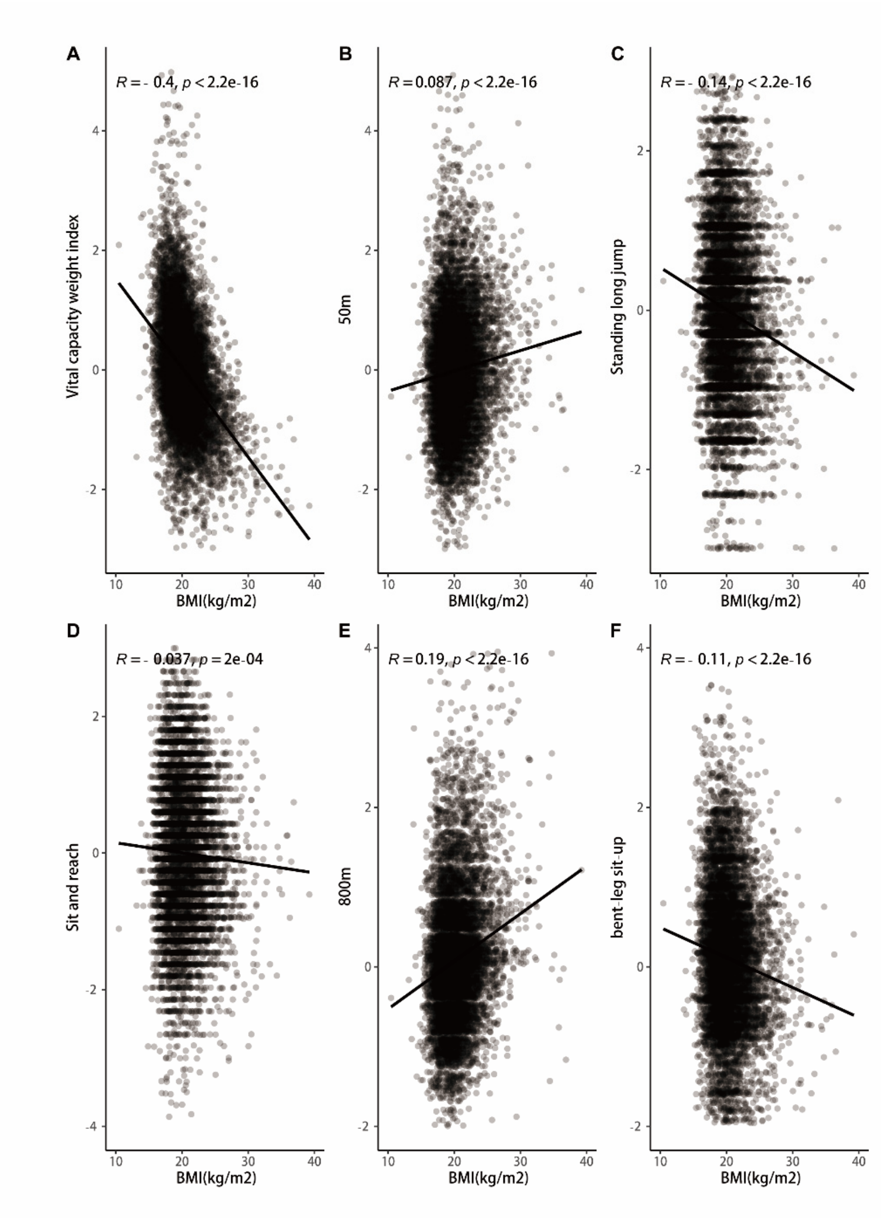

Supplementary Figure S2. Correlations between body mass index (BMI) and standardized physical fitness indicators in female students. Data for each fitness indicator were standardized (z-scores; mean = 0, SD = 1). Pearson correlation coefficients (r) and p-values are displayed for each BMI-indicator pair.
