# Supplementary material for: Association Between Body Mass Index and Physical Fitness Among University Students in a Sub-Plateau Region of Gansu Province, China: A Cross-Sectional Study
Source: Life (Basel). 2026 Jul 26;16(8):1235. doi: 10.3390/life16081235 (PMC13514759; doi:10.3390/life16081235)
Supplement: Supplementary file 1 [file life-16-01235-s001.zip › life-4378054-supplementary-Tables S1¿CS4.pdf]

Supplementary Table S1. Full pairwise comparison p-values for male students (42 comparisons total, aligned with Table 4)

Test Items	1 vs. 2	1 vs. 3	1 vs. 4	2 vs. 3	2 vs. 4	3 vs. 4
Vital capacity (ml)	<0.001 / <0.001 / <0.001	<0.001 / <0.001 / <0.001	<0.001 / <0.001 / <0.001	<0.001 / <0.001 / <0.001	<0.001 / 0.0002 / <0.001	0.071 / 1.000 / 0.076
VCWI (ml/kg)	<0.001 / <0.001 / <0.001	<0.001 / <0.001 / <0.001	<0.001 / <0.001 / <0.001	<0.001 / <0.001 / <0.001	<0.001 / <0.001 / <0.001	<0.001 / <0.001 / <0.001
50m (s)	0.880 / 1.000 / 0.880	<0.001 / <0.001 / <0.001	<0.001 / <0.001 / <0.001	<0.001 / <0.001 / <0.001	<0.001 / <0.001 / <0.001	<0.001 / <0.001 / <0.001
Standing long jump (cm)	0.044 / 1.000 / 0.050	<0.001 / <0.001 / <0.001	<0.001 / <0.001 / <0.001	<0.001 / <0.001 / <0.001	<0.001 / <0.001 / <0.001	<0.001 / <0.001 / <0.001
Sit and reach (cm)	0.0003 / 0.012 / 0.0004	0.028 / 1.000 / 0.033	0.048 / 1.000 / 0.053	0.383 / 1.000 / 0.402	<0.001 / 0.003 / <0.001	0.001 / 0.042 / 0.001
1000-m (s)	0.529 / 1.000 / 0.542	<0.001 / <0.001 / <0.001	<0.001 / <0.001 / <0.001	<0.001 / <0.001 / <0.001	<0.001 / <0.001 / <0.001	<0.001 / <0.001 / <0.001
Pull-up	<0.001 / 0.00001 / <0.001	<0.001 / <0.001 / <0.001	<0.001 / <0.001 / <0.001	<0.001 / <0.001 / <0.001	<0.001 / <0.001 / <0.001	0.0003 / 0.012 / 0.0004

Note: Group 1 = Underweight, Group 2 = Normal, Group 3 = Overweight, Group 4 = Obesity. Each cell presents results in the order of uncorrected p-value, Bonferroni-adjusted p-value, and Benjamini-Hochberg FDR-adjusted p-value (both corrections applied across all 42 pairwise comparisons per sex stratum; p-values calculated using R's p.adjust() function).

Supplementary Table S2. Full pairwise comparison p-values for female students (42 comparisons total, aligned with Table 5)

Test Items	1 vs. 2	1 vs. 3	1 vs. 4	2 vs. 3	2 vs. 4	3 vs. 4
Vital capacity (ml)	<0.001 / <0.001 / <0.001	<0.001 / <0.001 / <0.001	0.0003 / 0.012 / 0.001	<0.001 / 0.0001 / <0.001	0.230 / 1.000 / 0.268	0.549 / 1.000 / 0.606
VCWI (ml/kg)	<0.001 / <0.001 / <0.001	<0.001 / <0.001 / <0.001	<0.001 / <0.001 / <0.001	<0.001 / <0.001 / <0.001	<0.001 / <0.001 / <0.001	<0.001 / <0.001 / <0.001
50m (s)	0.627 / 1.000 / 0.675	<0.001 / <0.001 / <0.001	0.002 / 0.087 / 0.003	<0.001 / <0.001 / <0.001	0.003 / 0.110 / 0.004	0.327 / 1.000 / 0.371
Standing long jump (cm)	0.042 / 1.000 / 0.053	<0.001 / <0.001 / <0.001	<0.001 / <0.001 / <0.001	<0.001 / <0.001 / <0.001	<0.001 / 0.0004 / <0.001	0.025 / 1.000 / 0.033
Sit and reach (cm)	<0.001 / 0.004 / <0.001	0.167 / 1.000 / 0.206	0.001 / 0.053 / 0.002	0.0003 / 0.014 / 0.001	<0.001 / 0.002 / <0.001	0.012 / 0.492 / 0.016
800-m (s)	0.214 / 1.000 / 0.257	<0.001 / <0.001 / <0.001	<0.001 / <0.001 / <0.001	<0.001 / <0.001 / <0.001	<0.001 / <0.001 / <0.001	0.003 / 0.115 / 0.004
Bent-leg sit up	0.976 / 1.000 / 0.976	0.956 / 1.000 / 0.976	0.002 / 0.069 / 0.003	0.966 / 1.000 / 0.976	0.001 / 0.063 / 0.003	0.003 / 0.125 / 0.004

Note: Group 1 = Underweight, Group 2 = Normal, Group 3 = Overweight, Group 4 = Obesity. Each cell presents results in the order of uncorrected p-value, Bonferroni-adjusted p-value, and Benjamini-Hochberg FDR-adjusted p-value (both corrections applied across all 42 pairwise comparisons per sex stratum; p-values calculated using R's p.adjust() function).

Supplementary Table S3. Pearson correlation coefficients (r) between BMI and individual fitness indicators, stratified by sex

Test Item	Male (n = 4,813)		Female (n = 9,931)	
	**r**	*p*-value	**r**	*p*-value
VCWI (mL/kg)	**-0.47**	<0.001	**-0.40**	<0.001
50-m sprint (s)	**0.19**	<0.001	**0.087**	<0.001
Sit-and-reach (cm)	**-0.032**	0.025	**-0.037**	<0.001
1000-m (male) / 800-m (female) run (s)	**0.29**	<0.001	**0.19**	<0.001
Standing long jump (cm)	**-0.22**	<0.001	**-0.14**	<0.001
Pull-ups (male) / Bent-leg sit-ups (female)	**-0.30**	<0.001	**-0.11**	<0.001

Note: VCWI = weight-normalized vital capacity index. All p-values are two-tailed. **Bold values** indicate correlation coefficients extracted from the scatter plot matrices (Figures S1 and S2). BMI showed the strongest correlation with VCWI in both sexes and the weakest correlation with sit-and-reach flexibility. All correlations were statistically significant except for sit-and-reach in males ($p = 0.025$), which was nominally significant but negligible in magnitude.

Supplementary Table S4. Partial correlation coefficients between BMI and raw vital capacity (VC), controlling for height and age, stratified by sex

Sex	Control Variables	**r_partial (BMI vs. raw VC)**	df	*p* -value	Interpretation
Male	Height, Age	**0.208**	4,809	<0.001	BMI positively correlated with raw VC after adjusting for height and age
Female	Height, Age	**0.175**	9,927	<0.001	BMI positively correlated with raw VC after adjusting for height and age

Note: r_partial = partial correlation coefficient; df = degrees of freedom; VC = vital capacity. All p-values are two-tailed. **Bold values** indicate correlation coefficients extracted from the scatter plot matrices (Figures S1 and S2). Control variables (height and age) were included to minimize the mathematical coupling effect between BMI and VCWI (which shares body weight in its denominator), allowing the assessment of whether the inverse BMI–VCWI association reflects a genuine decline in weight-normalized pulmonary function or a purely statistical artifact. The positive partial correlations indicate that after removing the confounding effects of height and age, higher BMI is associated with higher raw VC, confirming that the observed inverse BMI–VCWI gradient is driven by weight normalization rather than by a biologically negative relationship between adiposity and lung volume per se.
